# MCT8 Deficiency: The Road to Therapies for a Rare Disease

**DOI:** 10.3389/fnins.2020.00380

**Published:** 2020-04-28

**Authors:** Carmen Grijota-Martínez, Soledad Bárez-López, David Gómez-Andrés, Ana Guadaño-Ferraz

**Affiliations:** ^1^Department of Endocrine and Nervous System Pathophysiology, Instituto de Investigaciones Biomédicas Alberto Sols, Consejo Superior de Investigaciones Científicas (CSIC), Universidad Autónoma de Madrid (UAM), Madrid, Spain; ^2^Center for Biomedical Research on Rare Diseases (Ciberer), Instituto de Salud Carlos III, Madrid, Spain; ^3^Department of Cell Biology, Faculty of Biology, Universidad Complutense de Madrid, Madrid, Spain; ^4^Translational Health Sciences, Dorothy Hodgkin Building, University of Bristol, Bristol, United Kingdom; ^5^Pediatric Neurology, Vall d’Hebron University Hospital and VHIR (Euro-NMD, ERN-RND), Barcelona, Spain

**Keywords:** thyroid hormones, brain, neurodevelopment, MCT8, DITPA, sobetirome, TRIAC

## Abstract

Allan-Herndon-Dudley syndrome is a rare disease caused by inactivating mutations in the *SLC16A2* gene, which encodes the monocarboxylate transporter 8 (MCT8), a transmembrane transporter specific for thyroid hormones (T3 and T4). Lack of MCT8 function produces serious neurological disturbances, most likely due to impaired transport of thyroid hormones across brain barriers during development resulting in severe brain hypothyroidism. Patients also suffer from thyrotoxicity in other organs due to the presence of a high concentration of T3 in the serum. An effective therapeutic strategy should restore thyroid hormone serum levels (both T3 and T4) and should address MCT8 transporter deficiency in brain barriers and neural cells, to enable the access of thyroid hormones to target neural cells. Unfortunately, targeted therapeutic options are currently scarce and their effect is limited to an improvement in the thyrotoxic state, with no sign of any neurological improvement. The use of thyroid hormone analogs such as TRIAC, DITPA, or sobetirome, that do not require MCT8 to cross cell membranes and whose controlled thyromimetic activity could potentially restore the normal function of the affected organs, are being explored to improve the cerebral availability of these analogs. Other strategies aiming to restore the transport of THs through MCT8 at the brain barriers and the cellular membranes include gene replacement therapy and the use of pharmacological chaperones. The design of an appropriate therapeutic strategy in combination with an early diagnosis (at prenatal stages), will be key aspects to improve the devastating alterations present in these patients.

## Introduction

Thyroid hormones (THs; T4 or thyroxine and T3 or triiodothyronine) are essential for the development and function of the CNS. An insufficient supply of THs during development can hinder essential processes such as neurogenesis, cell migration, synaptogenesis or myelinization. These neurological alterations range from very mild to very severe, and can be even irreversible, depending on the time and duration of the hormonal deficiency.

THs are synthesized in the thyroid gland, mostly as T4, and released by follicular cells to the circulation, reaching the different target tissues. Both genomic and non-genomic actions of THs have been described. The latter are carried out in the cytoplasm or plasma membrane by binding to receptors such as integrin *α*v*β*3 (Davis et al., 2016). On the other hand, in the genomic actions T3 binds to nuclear receptors (TR), regulating the transcription of target genes. The main isoforms of these TR are TR*α*1, TR*α*2, TR*β*1, and TR*β*2, which mediate different actions and whose expression varies throughout ontogeny and different tissues. The amount of the nuclear active hormone T3 is mainly regulated locally in the target tissues by the action of different deiodinase enzymes which activate (converting T4 into T3) or inactivate (deiodinating T4 and T3 into inactive metabolites) THs.

THs use transporter proteins located in cell membranes to reach the inside the cells (Hennemann et al., 2001). Some of the major transporters of THs include the monocarboxylate transporter 8 (MCT8), MCT10 and the solute carrier organic anion transporter 1C1 (OATP1C1), but only MCT8 has been identified as being specific to transport THs. MCT8 is encoded by the *SLC16A2* gene (Friesema et al., 2003) and the mutations that cause the inactivation of this transporter result in a rare X-linked disease known as Allan-Herndon-Dudley Syndrome (AHDS or MCT8 deficiency). AHDS (ORPHA:59) is an ultra-rare disorder with an estimated prevalence lower than 1 case per million. Orphanet suggested that 320 patients had been diagnosed within more than 130 families but these numbers are prone to raise given the increasing access to new generation sequencing technologies and the expanding spectrum of severity attributed to the disease (Remerand et al., 2019).

AHDS was described in 1944 (Allan et al., 1944) but its association to mutations in the *SLC16A2* gene was established in 2004 (Dumitrescu et al., 2004; Friesema et al., 2004). AHDS patients have a characteristic thyroid profile with elevated plasma T3 levels (peripheral hyperthyroidism), low T4 and normal or slightly elevated TSH with a free T3/T4 ratio greater than 0.75 (Dumitrescu et al., 2004; Friesema et al., 2004; Groeneweg et al., 2019; Remerand et al., 2019).

In addition, the phenotype of AHDS is characterized by severe paraparesis with hypotonia (with poor cephalic control being an early symptom), bradykinesia, spasticity and extrapyramidal manifestations (dystonia or dystonia with choreoathetosis) and moderate to severe intellectual disability with absence of language, associated with low weight and/or low muscle mass and in some cases, epilepsy (Remerand et al., 2019). In recent years, the increasing knowledge about this condition have promoted a growing number of patients that have defined a much broader phenotype, with the appearance of milder forms with motor and cognitive disability of lesser severity (Masnada et al., 2019; Remerand et al., 2019).

These alterations occur as a consequence of two phenomena. On the one hand, the excess of T3 in serum produces peripheral hyperthyroidism with muscle thyrotoxicosis that causes a generalized loss of muscle volume. On the other hand, the absence of MCT8 impairs the effective transport of THs to the CNS, most probably due to the lack of functional MCT8 in brain barriers (Ceballos et al., 2009; Iwayama et al., 2016; Vatine et al., 2017) leading to brain hypothyroidism (Dumitrescu et al., 2006; Trajkovic et al., 2007; López-Espíndola et al., 2014). Indeed, brain tissue necropsies of MCT8-deficient patients have revealed alterations compatible with brain hypothyroidism with anomalies in neuronal differentiation, myelinization and synaptogenesis present already from prenatal stages (López-Espíndola et al., 2014). Consistent with this, MCT8 has been found to be very abundant at the brain barriers in the human fetal brain (Wirth et al., 2009; López-Espíndola et al., 2019; Wilpert et al., 2020).

The rapid diagnosis is a key factor for the patients’ wellbeing. For instance, unlike other similar diseases, there is a marked prevalence of respiratory problems and kyphoscoliosis, so intensive monitoring and an adequate care is important to improve the survival and quality of life of patients. Moreover, due to the fact that brain alterations start during development, an early treatment, including prenatal treatment, takes special importance. Currently, targeted therapies to improve the neurological picture of patients are not available. As MCT8-deficient patients suffer from brain hypothyroidism in combination with peripheral hyperthyroidism, the design of an effective treatment is challenging. The aim of this manuscript is to critically review therapeutic approaches attempted so far both at the clinical level in patients and at the preclinical level in models of the disease to contribute to the development of future solutions for AHDS patients (summarized in Table 1).

**TABLE 1:** Summary of the existing clinical and preclinical therapeutic approaches for AHDS.

			Effects on	Effects on	Effects on	Effects on	
	Treatment	Model	serum TSH	serum T4	serum T3	brain	References
TH replacement therapies	LT4	Human	↓	↑	↑	Not observed	Namba et al., 2008; Papadimitriou et al., 2008
	
	LT4 + T3	Human	↓	↑	↑	Not observed	Biebermann et al., 2005; Zung et al., 2011
	
	LT4 + PTU	Human	↓	↑	↓	Not observed	Wemeau et al., 2008; Visser et al., 2013

TH analogs treatments	DITPA	Zebrafish	N/R	N/R	N/R	Prevents hypomyelination	Zada et al., 2014, 2016
		
		Mouse	↓	↓	↓	Only at high doses (0.01 mg/g BW)	Di Cosmo et al., 2009; Ferrara et al., 2015
		
		Human	↓	↑	↓	Not observed	Verge et al., 2012
	
	TRIAC	Zebrafish	N/R	N/R	N/R	Prevents and rescues hypomyelination	Zada et al., 2014, 2016
		
		Mouse	↓	↓	↓	Only at high doses (200–400 ng/g BW) and early treatment	Kersseboom et al., 2015; Bárez-López et al., 2016
		
		Human	↓	↓	↓	Under Investigation	Groeneweg et al., 2019
	
	Sobetirome	Mice	↓	↓	↓	Modulation of TH-target genes	Bárez-López et al., 2018

Other therapies	Gene replacement therapy	Zebrafish	N/R	N/R	N/R	Rescues hypomyelination	Zada et al., 2016
		
		Mice	N/R	N/R	**=**	Modulation of TH-target genes when MCT8 is replaced at brain barriers	Iwayama et al., 2016
	
	Pharmacological chaperones	*In vitro ex vivo*	N/A	N/A	N/A	N/A	Braun and Schweizer, 2015, 2017; Groeneweg et al., 2018

*↑, ↓, = : increased, decreased or not modified, respectively, versus non-treated patients or mouse model; N/R: not reported; N/A: not applicable.*

## Murine Models of the Disease

The development of targeted therapies in rare diseases depends on the availability of good animal models, both to unravel the mechanisms of disease and to carry out preclinical studies to evaluate therapeutic strategies. In the case of MCT8 deficiency, most of the knowledge about the mechanisms underlying the disease as well as the preclinical studies considered so far has arisen from using murine models of the syndrome. However, it has not been straightforward to obtain a suitable model and there is still ongoing work focused on the characterization of different animal models.

**FIGURE 1:**
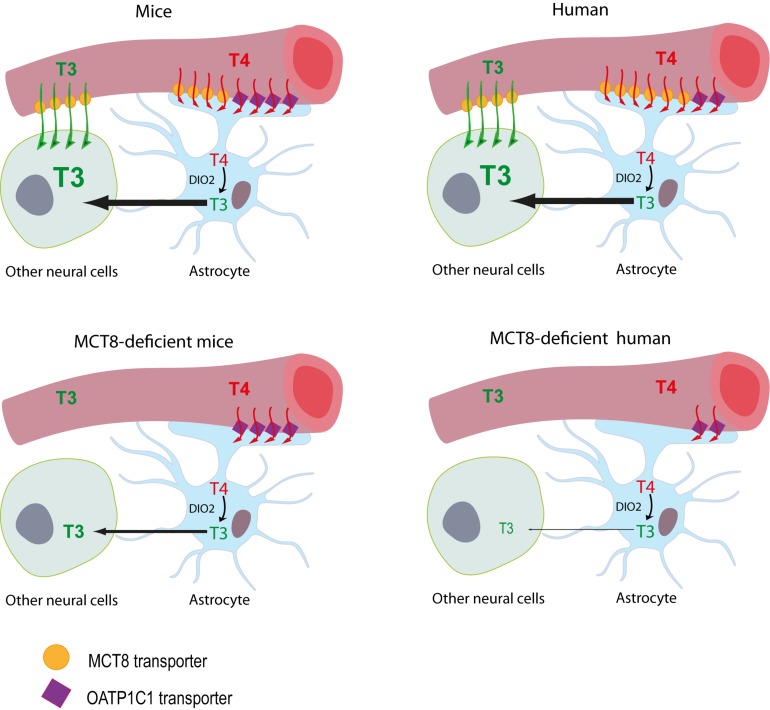
Proposed model illustrating the differences between MCT8-deficient mice and humans in thyroid hormone availability to neural cells. The model for T3 availability to the brain under normal conditions supports that brain T3 can access the target neural cells though two different routes: (1) directly from the circulation, with T3 crossing the BBB mainly via the MCT8 transporter into the extracellular fluid where it directly reaches the target neural cells, or (2) T3 can also be produced locally by DIO2 activity in the astrocytes from T4, which crosses the BBB directly into the astrocytes mainly through MCT8 in humans and through MCT8 and OATP1C1 in mice. In MCT8-deficient mice, the elevated DIO2 activity in the astrocytes converts the T4 available through OATP1C1 into T3, that is subsequently available to target neural cells, compensating for the lack of MCT8. In MCT8-deficient humans, this compensatory mechanism cannot take place as OATP1C1 is hardly present in the human BBB, preventing T4 entry to the brain and its subsequent conversion into T3.

Initially, mice deficient of the MCT8 transporter (*Mct8*KO mice) were proposed as a disease model. Although these mice showed a thyroid profile similar to that of the patients and relatively low levels of T3 in different brain areas (Dumitrescu et al., 2006; Trajkovic et al., 2007), brain histopathology studies did not reveal major defects and only subtle behavioral changes were found (Wirth et al., 2009). It was therefore suggested that these animals present some compensatory mechanism that prevents neurological damage. This compensatory mechanism is due to the complementary action of two proteins: the organic anion transporter OATP1C1 and the deiodinase type 2 (DIO2) enzyme. OATP1C1 transporter is codified by the *Slco1c1* gene, it transports mainly T4 and is expressed abundantly in rodent brain barriers, but to a much lesser extent in humans (Roberts et al., 2008; López-Espíndola et al., 2019). The entry of T4 to the brain through this transporter and the subsequent action of DIO2, which converts T4 into T3 in astrocytes and whose expression is increased in these mice, would provide a sufficient amount of the nuclear active hormone T3 in the mouse brain, exerting a compensatory mechanism for the lack of MCT8 (Figure 1). For this reason, two alternative models are currently being used; MCT8 and DIO2 *(Mct8/Dio2KO*) or MCT8 and OATP1C1 (*Mct8/Slc1c1*KO) -deficient mice, in which this compensatory mechanism is suppressed. These models present a thyroid profile similar to that of the patients in addition to several neuromotor alterations, offering a more suitable model (Mayerl et al., 2014; Bárez-López et al., 2019a).

In addition, other alternative models currently available include the use of pluripotent stem cells induced from human patients (Vatine et al., 2017) or the use of other organisms such as zebrafish (Vatine et al., 2013), chicken (Delbaere et al., 2017) or even Xenopus (Mughal et al., 2017). For the first time, this rich cataloge of models is providing the possibility of testing drugs in a fast and accurate way in preclinical stages in AHDS.

## Thyroid Hormone Replacement Therapies

As soon as it was found that the syndrome was due to mutations in a transporter of THs, and that it had an endocrine component with altered THs levels, treatments to intervene on the thyroid axis were considered. In *Mct8*KO mice, the injection of supraphysiological doses of levothyroxine (LT4) was able to reduce the increased expression of TRH mRNA in hypothalamic neurons, indicating that in the absence of MCT8 these neurons can respond to T4 treatment. In contrast, only a high dose of T3 was able to downregulate TSH levels in plasma with a modest reduction of TRH, suggesting an impaired uptake of circulating T3 (Trajkovic et al., 2007).

Based on this, in patients, a LT4 supplement alone or in combination with T3 was initially considered. This treatment suppressed TSH and restored T4; however, it further increased T3 serum levels, worsening the hyperthyroidism in extracerebral tissues, and did not improve the patient’s neurological impairments (Namba et al., 2008; Papadimitriou et al., 2008). After this first approximation, a block-replacement therapy consisting of the administration of propylthiouracil (PTU; an antithyroid drug that also inhibits type 1 deiodinase (DIO1), which transforms T4 into T3) together with LT4, decreased endogenous production of THs and prevented the conversion of LT4 into T3. This therapy normalized TSH levels and significantly decreased serum T3, which was reflected in an improvement in body mass index and a reduction in heart rate. However, none of the patients experienced any improvement at the psychomotor level (Wemeau et al., 2008; Visser et al., 2013).

## Treatment With Thyroid Hormone Analogs

The search for T3 analogs that do not require MCT8 to cross the cell membranes is one of the main strategies in the search for an effective therapy. 3,5-diiodo-thyropropionic acid (DITPA) is a T3 analog that binds with similar affinity to the different TR isoforms, although with much lower affinity than T3 (Pennock et al., 1992). Despite this, it has been shown to have inotropic thyromimetic actions but maintaining a low metabolic activity (Pennock et al., 1992; Moreno et al., 2008). In zebrafish, treatment with DITPA was able to prevent hypomyelination in Mct8-deficient embryos but did not fully recovered hypomyelination present in the larvae (Zada et al., 2014, 2016). The administration of this compound to MCT8-deficient mice was able to normalize T3 and TSH while it further decreased T4 levels in serum, ameliorating metabolic parameters related to the hypermetabolism of *Mct8*KO mice. However, improvements in brain hypothyroidism could only be observed when using high doses of DITPA (Di Cosmo et al., 2009; Ferrara et al., 2015). The administration of DITPA in four patients normalized T3 and TSH and, unlike in mice, increased the levels of T4 to the lower normal range in serum, decreasing also the cardiac rhythm. However, no improvement, or very small, was found in the neurological performance of these patients when they were assessed with the Bayley-III scale (Verge et al., 2012).

Another T3 analog that has received special attention is sobetirome (also called GC-1), a compound that has been used in numerous animal studies (Hartley et al., 2017) as well as in clinical trials for patients with dyslipidemias. Sobetirome binds to TRs with the same affinity for TRβ1 as T3 but 4–10 times less affinity than T3 for TRα1 (Chiellini et al., 1998). In 2018 we published the first study evaluating its action in the absence of MCT8 after intraperitoneal administration to *Mct8/Dio2*KO mice (Bárez-López et al., 2018). An increase in the content of sobetirome was detected in the brain of the treated animals, demonstrating that it was able to cross the blood-brain barrier of mice in the absence of MCT8. Most importantly, we provided evidence of sobetirome action in brain, regulating the expression of T3 target genes, since it was able to partially recover and even normalize the expression of some genes that are altered in *Mct8/Dio2*KO animals.

A third and promising alternative is 3,5,3’-triiodothyroacetic acid (TRIAC), a natural metabolite of THs. This compound arises as one of the potential options for the treatment of patients since *in vitro* studies have shown that it can bind the TR with the same affinity as T3 for the isoform TRα and with greater affinity for TRβ (Takeda et al., 1995; Messier and Langlois, 2000), that it can exert thyromimetic actions, and that can access the inside of the cell in the absence of MCT8 (Horn et al., 2013; Kersseboom et al., 2015). Moreover, treatment with TRIAC in zebrafish was able to prevent and rescue hypomyelination in Mct8-deficient embryos and larvae, respectively (Zada et al., 2014, 2016). The first study evaluating the *in vivo* action of TRIAC in MCT8 deficiency was carried out in *Mct8/Slco1c1*KO mice treated with high doses of TRIAC (200-400 ng per gram of weight per day) during early postnatal stages (from postnatal day 1 (P1) to P12). These studies revealed that at high doses TRIAC is able to prevent brain alterations in the absence of MCT8 (Kersseboom et al., 2015). To get a better understanding on the potential of this analog in the clinical practice, we assessed the effects of therapeutic doses of TRIAC at juvenile stages. *Mct8*KO mice were treated with of 30 ng of TRIAC per gram of weight per day (similar to the dose used in patients) in the drinking water from P21 to P30. This treatment decreased serum levels of both T3 and T4, improving the peripheral hyperthyroidism. However, TRIAC content in the brain did not increase after treatment, and therefore no thyromimetic actions could be observed in this organ. Moreover, we also found that the decrease in T4 plasma levels worsened brain hypothyroidism by further decreasing the brain T3 content and the expression of some target genes (Bárez-López et al., 2016). This highlights the importance of considering therapeutic approaches that do not further decrease serum T4.

Currently, alternative delivery pathways are being explored in order to increase TRIAC content in the brain with the final aim of ameliorating the brain hypothyroidism. In a recent study we administered TRIAC directly into the brain of MCT8-deficient animals through a catheter inserted into the brain lateral ventricle (Bárez-López et al., 2019b). We found that this treatment did not affect T3 or T4 levels in the serum, thus did not aggravate the brain hypothyroidism. Moreover, even though TRIAC content increased in the brain after treatment, this increase was not enough to mediate the expression of T3 target genes. Further studies will be necessary to finally determine if TRIAC serves as a T3 analog mediating brain effects of THs *in vivo*.

Due to the extensive clinical experience with TRIAC, that has demonstrated its ability to inhibit TSH secretion and therefore decrease the production of endogenous THs, TRIAC has been used in a phase I clinical trial evaluating its effectiveness and safety for treatment of peripheral thyrotoxicosis in MCT8-deficient patients, both pediatric and adult (Groeneweg et al., 2019). In accordance with the results found in the animal model, treatment with 38.3 μg of TRIAC per kg of body weight per day reduced serum T3 levels to the normal range and further decreased serum T4 levels. This study has shown that TRIAC is safe and effective in controlling peripheral thyrotoxicosis improving the altered weight, heart rate and blood pressure. However, it is important to point out that reduced plasma T4 levels could have potential adverse consequences on the brain, as was found in systemically TRIAC-treated *Mct8*KO mice. Having evaluated the effects of TRIAC on peripheral thyrotoxicosis and showing encouraging effects (a trend toward improvement) in gross motor function when children are treated before 4 year of age, the clinical study is currently in phase II to evaluate the action of this analog on neurological symptoms in pediatric patients (NCT02396459).

## Other Therapies for MCT8 Deficiency

In parallel to the use of T3 analogs, other possibilities such as gene replacement therapy and the use of chemical chaperones are also currently being explored. In order to improve brain alterations by gene therapy in mice, MCT8 was administered using adeno-associated virus (AAV) as a vector with the intention of restoring MCT8 expression in the brain. For this purpose, AAV9 (which is transduced in the brain with high efficiency) expressing MCT8 was injected to MCT8-deficient mice at day P1 either intravenously or directly into the brain intracerebroventricularly. The results showed that intravenous administration increased the content of T3 in the brain and recovered the expression of T3 target genes altered in MCT8 deficiency. Although intracerebroventricular administration drove higher expression of MCT8 in the brain than the intravenous delivery, there was no increase in the content of T3 or in the expression of T3 target genes. This could be explained because, unlike the intracerebroventricular route, the intravenous delivery was able to restore MCT8 expression in the choroid plexus demonstrating that for a successful gene replacement therapy, it is essential to restore MCT8 in brain barriers to allow entry of THs into the brain (Iwayama et al., 2016). In zebrafish, the injection of a genetic construct driving Mct8 expression in vascular endothelial cells into Mct8-deficient embryos was able to recover hypomyelination in 10 days post fertilization larvae but not in 3 days post fertilization embryos. This suggests that genetic treatment can be beneficial even after brain damage has occurred and offers a potential treatment for MCT8-deficient patients (Zada et al., 2016).

A different approximation is the use of pharmacological chaperones that allow the conformational stabilization of mutated proteins and assist in their translocation to the plasma membrane. It is believed that this approach would be more effective for those mutations in *SLC16A2* that affect the stability of the protein and its intracellular traffic. A recent work using stable overexpression cell models demonstrated that the use of pharmacological chaperones can restore MCT8 in cell membranes and mediate T3 transport in certain types of *SLC16A2* mutations (Braun and Schweizer, 2015, 2017). However, similar studies have subsequently been carried out *ex vivo* on fibroblasts derived from MCT8-deficient patients without functionally restoring MCT8 in cell membranes, and thus demonstrating that the ability of pharmacological chaperones to rescue MCT8 function depends on the cell type used for the assays (Groeneweg et al., 2018).

## Conclusion

The complex nature of MCT8 deficiency presents certain difficulties when designing appropriate therapeutic strategies. A suitable therapy should ameliorate the peripheral hyperthyroidism while improving the neurological defects. So far, several therapeutic approaches have been successful in improving the peripheral hyperthyroidism in patients. These include a block-replacement therapy with LT4 in combination with PTU and the T3 analogs DITPA and TRIAC. Treatment with TRIAC further decreases T4 serum levels unlike DITPA and LT4 + PTU treatments. Due to the potential effects that T4 (through local conversion into T3) might be mediating in the brain, an ultimate therapeutic approach should not further decrease but restore serum T4 levels.

The big challenge ahead of us is the improvement of the neurological alterations in patients. To tackle this, several approaches that aim to overcome the limitation of the brain barriers are currently being explored. The use of alternative delivery pathways that can increase the content of T3 analogs in the brain offers the possibility of enhancing the potential action of these analogs in the brain, as long as they are indeed effective in mediating TH-like actions *in vivo*. The use of the sobetirome, gene replacement therapy and pharmaceutical chaperones offers promising alternative therapies that require further characterization. Moreover, as an early treatment is crucial, the use of T3 analogs that can cross the placental barrier such as TRIAC (Nicolini et al., 1996) or DITPA (Ferrara et al., 2014) or the direct *in utero* administration of potential therapeutic agents, must be considered.

Due to the great advances in preclinical stages of potential therapies for AHDS, we believe that studies devoted to enhance our understanding about the role of MCT8 in development and the complex changes in the CNS related with this disorder are particularly urgent in order to proceed with an optimal translation of the different therapeutic approaches to patients.

## Author Contributions

CG-M, SB-L, DG-A, and AG-F have contributed in the drafting of the article and have revised it critically for important intellectual content and for the final approval of the submitted version. CG-M and SB-L have designed the figures.

## Conflict of Interest

The authors declare that the research was conducted in the absence of any commercial or financial relationships that could be construed as a potential conflict of interest.
